# Immune checkpoint inhibitor‐associated interstitial lung diseases correlate with better prognosis in patients with advanced non‐small‐cell lung cancer

**DOI:** 10.1111/1759-7714.13364

**Published:** 2020-02-25

**Authors:** Teppei Sugano, Masahiro Seike, Yoshinobu Saito, Takeru Kashiwada, Yasuhiro Terasaki, Natsuki Takano, Kakeru Hisakane, Satoshi Takahashi, Toru Tanaka, Susumu Takeuchi, Akihiko Miyanaga, Yuji Minegishi, Rintaro Noro, Kaoru Kubota, Akihiko Gemma

**Affiliations:** ^1^ Department of Pulmonary Medicine and Oncology, Graduate School of Medicine Nippon Medical School Tokyo Japan; ^2^ Department of Analytic Human Pathology, Graduate School of Medicine Nippon Medical School Tokyo Japan

**Keywords:** Immune check inhibitor, immune‐related adverse events, interstitial lung disease, prognosis

## Abstract

**Background:**

Interstitial lung disease (ILD) induced by immune checkpoint inhibitors (ICIs) is a potentially life‐threatening adverse event. The purpose of this study was to evaluate whether the development of immune‐related adverse events (irAEs), especially ILD, was associated with treatment efficacy and to research the features and risk factors of ILD in advanced non‐small cell lung cancer (NSCLC).

**Methods:**

Between December 2015 and November 2018, 130 advanced NSCLC patients were treated with nivolumab, pembrolizumab or atezolizumab. The patients were categorized into two groups (irAEs group or non‐irAEs group). Subsequently, we divided the irAEs group into two groups based on the incidence of ILD (ILD group and irAEs‐non‐ILD group). Treatment efficacy and the characteristics of ILD were evaluated.

**Results:**

A total of 39 (30%) patients developed irAEs. ILD was observed in 16 (12%) patients. Patients with ILD had a higher objective response rate (ORR) compared with irAEs‐non‐ILD patients and non‐irAEs patients (63%, 43% and 22%, respectively). Median progression‐free survival (mPFS) was 15.9 months in ILD patients, 5.4 months in irAEs‐non‐ILD patients and 3.3 months in non‐irAEs patients (log‐rank test, *P* = 0.033). Pre‐existing interstitial pneumonia (IP) was an independent risk factor for ILD‐induced ICIs (odds ratio [OR] 14.7; 95% confidence interval [CI]: 2.16–99.6, *P* = 0.006).

**Conclusions:**

ORR and PFS were significantly better in ILD patients than in irAEs‐non‐ILD and non‐irAEs patients. Pre‐existing history of IP was an independent risk factor for ILD‐induced ICIs.

## Introduction

Blockade of the programmed cell death protein‐1 (PD‐1) pathway has been established as a novel standard treatment for patients with a variety of malignancies.^1^ Nivolumab and pembrolizumab are full human IgG4 PD‐1 checkpoint inhibitor antibodies that selectively target programmed death (PD)‐1 receptors, PD ligand 1 and 2 (PD‐L1 and PD‐L2). In the CheckMate 017 and 057 trials, nivolumab prolonged overall survival (OS) compared with docetaxel in patients with previously treated non‐small cell lung cancer (NSCLC).^2^, ^3^ In the KEYNOTE 024 trial, pembrolizumab was significantly associated with longer OS than platinum‐doublet chemotherapy in patients with previously untreated advanced NSCLC with strongly positive tumor PD‐L1 expression.^4^ Atezolizumab is a monoclonal antibody that targets PD‐L1. In the OAK study, atezolizumab improved the OS compared with docetaxel.^5^ However, immune checkpoint inhibitors (ICIs) can cause inflammatory reactions, termed immune‐related adverse events (irAEs), such as skin reactions, thyroid dysfunction, interstitial lung disease (ILD), type I diabetes, and hypophysitis.^2^, ^3^, ^4^, ^5^ IrAEs are different from the adverse effects induced by conventional cytotoxic agents and in some cases, they require systemic immunosuppressive treatment. Among irAEs, ILD is known to lead to serious lung injury or life‐threatening complications.^2^, ^3^, ^4^, ^5^

Recently, several studies have reported a positive correlation between irAEs and improved clinical outcomes.^6^, ^7^ However, an association between ILD and the efficacy of ICIs has not been fully investigated. The aim of this study was to evaluate the relationship between the development of ILD and clinical efficacy and to clarify the features and risk factors of ILD for patients with advanced NSCLC treated with ICIs.

## Methods

### Patients and data collection

We retrospectively reviewed clinical data including prior chemotherapy, radiation treatment, response to therapy, and adverse events from medical charts. The objective tumor response was evaluated using the Response Evaluation Criteria for Solid Tumors (RECIST ver. 1.1).^8^ Progression‐free survival (PFS) was calculated from the first day of treatment to documented disease progression or death due to any cause. IrAEs were defined according to previous studies,^2^, ^3^, ^4^, ^5^ and ILD was diagnosed based on clinical, physiological, and chest computerized tomography (CT) scan findings. Grades of irAEs were evaluated based on the Common Terminology Criteria for Adverse Events (CTCAE), version 4.0. The patients were divided into two groups based on the incidence of irAEs: those with irAEs (irAEs group) or those without irAEs (non‐irAEs group). Patients in the irAEs group were further categorized into two groups: those with ILD (ILD group) and those without ILD (irAEs‐non‐ILD group). Subsequently, we evaluated the overall response rate (ORR) and median progression‐free survival (mPFS) in each group.

### Statistical analysis

The cutoff date was 28 February 2019. Chi‐squared tests were used to determine differences between groups. Survival curves were estimated by the Kaplan‐Meier method and compared by the log‐rank test. *P*‐values were estimated by comparisons between three groups (ILD, irAEs‐non‐ILD, and non‐irAEs). Univariate and multivariate analyses were carried out using the Cox regression model. Logistic regression analysis was used to identify potential risk factors of ILD. The results were expressed as the odds ratio (OR) with 95% confidence intervals (CIs). A *P*‐value of <0.05 indicated statistical significance. All statistical analyses were performed with EZR (Saitama Medical Centre, Jichi Medical University, Saitama, Japan), a graphical user interface for R (R Foundation for Statistical Computing, Vienna, Austria). Specifically, it is a modified version of R Commander designed to add statistical functions frequently used in biostatistics.^9^ The study was approved by the institutional review board of Nippon Medical School (IRB No. 30‐11‐1041).

## Results

### Patient characteristics

Between December 2015 and November 2018, 130 advanced NSCLC patients who had received nivolumab (3 mg/kg, every two weeks), pembrolizumab (200 mg/bodyweight, every three weeks), or atezolizumab (1200 mg/bodyweight, every three weeks) monotherapy at Nippon Medical School Hospital were enrolled. Patient characteristics are summarized in Table 1: ≥65 years/<65 years, 87/43; male/female, 98/32; Eastern Cooperative Oncology Group performance status (ECOG PS) of 0–1/≥ 2, 99/31; and squamous/adenocarcinoma/other, 52/64/14. Epidermal growth factor receptor (*EGFR*) mutant NSCLC was diagnosed in 14 patients. A total of 38 patients (29%) had received prior thoracic radiation therapy and 103 (78%) had undergone prior chemotherapy. Nivolumab, pembrolizumab, or atezolizumab were administrated to 67, 45, and 18 patients, respectively. The PD‐L1 expression showed 0/1–50/≥50/not evaluated, 17/27/39/47. No patients received two or more ICIs.

**Table 1:** Clinical characteristics of patients

Characteristics	Total (*n* = 130), *n* (%)	ILD group (*n* = 16), *n* (%)	irAEs‐non‐ILD group (*n* = 23), *n* (%)	Non‐irAEs group (*n* = 91), *n* (%)
Age (years)				
≥65	87 (67)	14 (88)	13 (57)	60 (66)
<65	43 (33)	2 (12)	10 (43)	31 (34)
Gender				
Male	98 (75)	11 (69)	18 (78)	69 (76)
Female	32 (25)	5 (31)	5 (22)	22 (24)
Smoking status				
Never	22 (17)	2 (12)	4 (17)	16 (18)
Former	91 (70)	12 (76)	14 (61)	65 (71)
Current	17 (13)	2 (12)	5 (22)	10 (11)
ECOG PS				
0–1	99 (76)	12 (76)	21 (91)	66 (73)
≥2	31 (24)	4 (24)	2 (9)	25 (27)
Histopathology				
Squamous	52 (40)	8 (50)	11 (48)	33 (37)
Adenocarcinoma	64 (49)	6 (38)	10 (43)	48 (53)
Other	14 (11)	2 (12)	2 (9)	10 (10)
*EGFR* status				
Wild‐type	113 (87)	14 (98)	22 (99)	77 (84)
Mutant	14 (11)	1 (1)	0 (0)	13 (15)
NE	3 (2)	1 (1)	1 (1)	1 (1)
Prior thoracic RT				
No	92 (71)	12 (76)	15 (65)	65 (72)
Yes	38 (29)	4 (24)	8 (35)	26 (28)
Prior chemotherapy				
No	27 (22)	6 (38)	6 (26)	15 (17)
Yes	103 (78)	10 (62)	17 (74)	76 (83)
PD‐1 inhibitor				
Nivolumab	67 (51)	7 (44)	14 (61)	46 (50)
Pembrolizumab	45 (34)	6 (38)	6 (26)	33 (35)
Atezolizumab	18 (15)	3 (18)	3 (13)	12 (15)
PD‐L1 expression				
0	17 (13)	0 (0)	2 (9)	15 (16)
1–50	27 (21)	2 (12)	3 (13)	22 (24)
≥50	39 (30)	7 (44)	6 (26)	27 (29)
NE	47 (36)	7 (44)	12 (52)	29 (31)

ECOG PS, European cooperative oncology group performance status; EGFR, epidermal growth factor receptor; ILD, interstitial lung disease; irAEs, immune related adverse events; NE, not evaluated; PD‐1, programmed death‐1; PD‐L1, programmed death‐1 ligand; RT, radiotherapy.

### Efficacy in all patients

**Figure 1:**
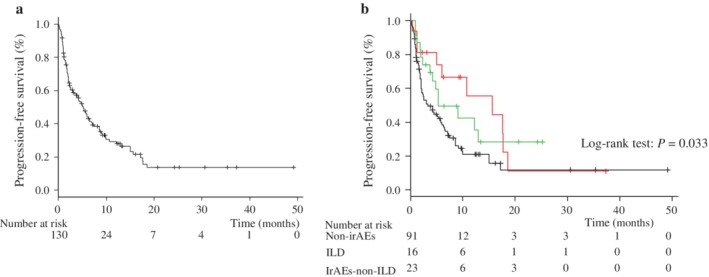
Rate of progression‐free survival (PFS) in the study population. Kaplan‐Meier curves are shown for progression‐free survival. (**a**) 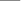 Median PFS for overall patients. (**b**) median PFS, 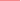 line; ILD, 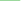 line; irAEs‐non‐ILD, 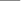 line; non‐irAEs. ILD, interstitial lung disease; irAEs, immune‐related adverse events; NR, not reached.

At the time of the data cutoff point, 26 patients continued ICI treatment. The most common reason for discontinuation was disease progression. The ORR was 30%: complete response (CR) was observed in 0 (0%), partial response (PR) in 39 (30%), stable disease (SD) in 36 (27%), and progressive disease (PD) in 40 patients (31%) (Table S1). The Kaplan‐Meier curve for PFS in all patients is shown in Figure 1a. A total of 95 PFS events (72%) occurred during the study period. The mPFS was 5.3 months (95% CI: 3.1 to 6.7). The mPFS of the untreated group was 9.3 months (95% CI: 3.8 to 18.8), and 4.9 months in the previously treated group (95% CI: 2.4 to 6.1) (Fig S1a,b).

### Categorization into ILD, irAEs‐non‐ILD and non‐irAEs groups

A total of 39 (30%) patients developed irAEs of any grade. Table S2 shows a summary of irAEs: 16 (12%) had ILD, nine (6.8%) had hypothyroidism, five (3.8%) had a skin reaction, three (2.2%) had nephrotoxicity, and two (1.5%) had encephalitis.

Subsequently, we divided irAE patients into two groups based on the incidence of ILD: those with ILD (ILD group) and those without ILD (irAEs‐non‐ILD group). Among 16 patients, only one patient developed both ILD and other (ocular myasthenia gravis).

Table 1 shows a comparison of the patient characteristics between the ILD, irAEs‐non‐ILD, and non‐irAEs groups.

Patients with ILD had a higher ORR compared with irAEs‐non‐ILD patients and non‐irAEs patients (63% vs. 43% and 21%, respectively) (Table S1). The mPFS among ILD patients was longer (15.9 months; 95% CI, 5.0 to 18.8, *P* = 0.033) compared with irAEs‐non‐ILD patients (5.4 months; 95% CI, 3.8 to not reached) and non‐irAEs patients (3.3 months; 95% CI, 2.1 to 5.9) (Fig 1b). The PFS of untreated patients is shown in Fig S2a. The mPFS of ILD, irAEs‐non‐ILD, and non‐irAEs groups was 15.9 months, not reached (NR) and 8.7 months, respectively (*P* = 0.09). The PFS of previously treated patients is shown in Fig S2b. The mPFS of ILD, irAEs‐non‐ILD, and non‐irAEs patients was 10.9, 5.4, and 3.1 months, respectively (*P* = 0.17). Among ILD and non‐irAEs patients, univariate and multivariate analyses were conducted. Univariate analysis revealed that nonsquamous, PS 0–1 and ILD indicated a better prognosis. Multivariate analysis also showed that nonsquamous, PS 0–1 and the incidence of ILD were independent predictors for better prognosis (Table 2 **)**.

**Table 2:** Cox proportional hazard regression analysis on progression‐free survival

	Univariate analysis			Multivariate analysis		
	Hazard ratio	95% CI	*P*‐value	Hazard ratio	95% CI	*P‐*value
Age, years						
<65 vs. ≥65	0.94	0.57–1.51	0.65			
Sex						
Male vs. female	1.21	0.72–2.02	0.45			
Histology						
Nonsquamous vs. squamous	1.72	1.08–2.72	**0.02**	1.67	1.05–2.63	**0.029**
Smoking history						
Never vs. ever	0.73	0.40–1.34	0.23			
PS						
0–1 vs. 2–4	2.76	1.67–4.55	**<0.001**	3.15	1.88–5.26	**<0.001**
Thoracic RT						
No vs. yes	0.77	0.47–1.26	0.29			
Treatment of ICIs						
Untreated vs. previously treated	1.23	0.69–2.21	0.47			
ILD						
Non‐irAEs vs. ILD	0.49	0.25–0.96	**0.04**	0.39	0.19–0.77	**0.007**

Bold values means *p* < 0.05. ICIs, immune checkpoint inhibitors; ILD, interstitial lung disease; irAEs, immune related adverse events; PS, performance status; RT, radiotherapy.

### Characteristics of ILD

**Figure 2:**
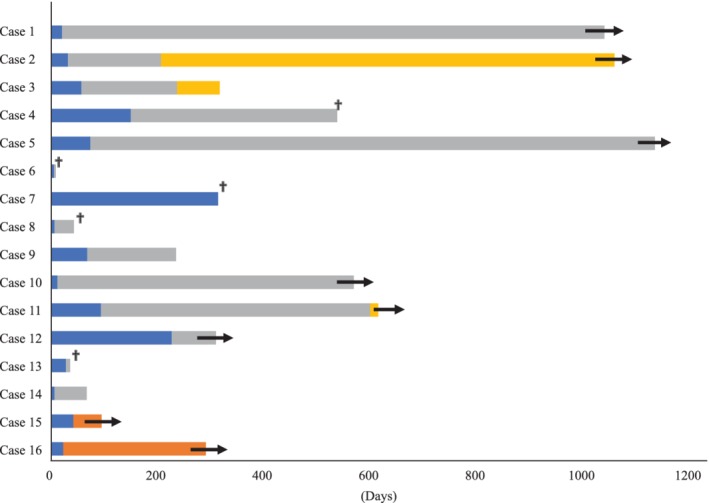
Duration of treatment in patients treated with ICIs. ICIs, immune checkpoint inhibitors; ILD, interstitial lung disease (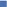 time to ILD), (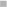 Off ICIs), (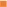
Continued ICIs), (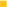
Another therapy), (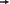
Ongoing), (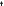 Death).

A total of 16 patients developed ILD. The incidence of ILD was 10% among patients treated with nivolumab, 13% with pembrolizumab, and 16% with atezolizumab. The clinical features of ILD are summarized in Table 3. The median time to the development of ILD was 28 days, with a wide range from six to 314 days. Any grade of ILD developed in 16 patients, of which three of 16 (18.8%) were grade 1, two of 16 (12.5%) were grade 2, eight of 16 (50.0%) were grade 3; and three of 16 (18.8%) were grade 5. The radiologic pattern of ILD was confirmed by two certificated respiratory physicians (TS and YS) and identified as organizing pneumonia (OP) in nine of 16 patients (56%), with two of 16 (13%) having diffuse alveolar damage (DAD), one of 16 (6%) having hypersensitivity pneumonia (HP), and four of 16 (25%) having not otherwise specified (NOS) (Fig S3). Among patients developing grade 5 ILD, two patients showed a DAD pattern and the other patient showed an OP pattern. Moreover, patients with the OP pattern tended to have a good prognosis compared with those with a non‐OP pattern (Fig S4). Five patients underwent transbronchial lung biopsy (TBLB). OP was observed in three of five TBLB specimens (60%), and cellular alveolitis with lymphocytic infiltration was observed in two of five (40%). Bronchoalveolar lavage fluid (BALF) was collected from consolidation or the GGO area from four patients. Lymphocytes were the predominant cell type in three of four patients. The CD4/CD8 ratio was examined by flow cytometric analysis and CD8 T cells were significantly elevated in three of four patients. Treatment of ILD was conducted in 12 of 16 (75.0%) patients: 10 patients received intravenous steroid pulse therapy and two patients received oral steroid therapy. Among those receiving steroid pulse treatment, five patients received immunosuppressant therapy with cyclophosphamide. ILD was improved or resolved in 13 patients. A total of 14 patients discontinued ICI treatment and long‐term tumor regression was observed during ICIs off periods (Fig 2). Seven of 16 patients had pre‐existing interstitial pneumonia (IP). Univariate and multivariate logistic regression analyses showed pre‐existing IP was an independent risk factor for ILD‐induced ICIs (multivariate logistic analysis: OR: 14.7; 95% CI: 2.16–99.6, *P* = 0.006) (Table 4 **)**.

**Table 3:** Clinical features of ILD

Patient	Age (years)	Gender	Histology	ICIs	Response	ILD Grade	Time to ILD (Days)	Pre‐existing IP	Radiologic pattern	Histopathological findings of ILD	Treatment	Outcome
Case 1	82	Male	Ad	Nivolumab	SD	3	21	No	OP	OP	Steroid pulse	Improved
Case 2	76	Male	Sq	Nivolumab	PR	3	32	No	OP	—	Steroid pulse Cyclophosphamide	Improved
Case 3	72	Female	Sq	Nivolumab	PR	3	57	Yes	OP	—	Steroid pulse Cyclophosphamide	Improved
Case 4	75	Male	Ad	Nivolumab	PR	3	150	No	OP	OP	Steroid pulse	Improved
Case 5	63	Female	Ad	Nivolumab	PR	1	74	No	OP	—	None	Improved
Case 6	61	Male	Sq	Nivolumab	NE	5	6	Yes	DAD	—	Steroid pulse Cyclophosphamide	Died
Case 7	74	Male	Sq	Nivolumab	PR	5	314	No	OP	—	Steroid pulse Cyclophosphamide	Died
Case 8	73	Male	Sq	Pembrolizumab	PD	3	7	No	HP	—	Steroid pulse	Improved
Case 9	82	Male	Pleomorphic	Pembrolizumab	PR	2	68	No	OP	OP	None	Improved
Case 10	69	Female	Sq	Pembrolizumab	PR	3	12	No	NOS	Cellular alveolitis	Steroid pulse	Improved
Case 11	68	Female	Ad	Pembrolizumab	PR	3	94	No	OP	Cellular alveolitis	Oral prednisolone 1 mg/kg	Improved
Case 12	80	Male	NSCLC	Pembrolizumab	PR	2	227	No	OP	—	Oral prednisolone 1 mg/kg	Improved
Case 13	80	Male	Sq	Pembrolizumab	NE	5	28	Yes	DAD	—	Steroid pulse Cyclophosphamide	Died
Case 14	70	Male	Ad	Atezolizumab	SD	3	7	Yes	NOS	—	Steroid half pulse	Improved
Case 15	75	Female	Ad	Atezolizumab	PD	1	42	No	NOS	—	None	Improved
Case 16	80	Male	Sq	Atezolizumab	PR	1	23	No	NOS	—	None	Improved

Ad, adeno; DAD, diffuse alveolar damage; GGO, ground‐glass opacities; ILD, interstitial lung disease; IP, interstitial pneumonia; NE; not evaluable; NOS, not otherwise specified; NSCLC, non‐small cell lung cancer; OP, organizing pneumonia; PR, partial response; SD, stable disease; Sq, squamous.

**Table 4:** Univariate and multivariate logistic regression analyses of patient characteristics and factors associated with potential risk factors for interstitial lung disease

	Univariate analysis			Multivariate analysis		
	ORs	95% CI	*P‐*value	ORs	95% CI	*P‐*value
Age, years						
<65 vs. ≥65	3.93	0.85–18.2	0.08	4.47	0.88–22.7	0.07
Sex						
Male vs. female	1.46	0.47–4.59	0.51	3.06	0.69–13.6	0.14
Histology						
Nonsquamous vs. squamous	1.59	0.56–4.55	0.39	0.81	0.23–2.85	0.75
Smoking history						
Never vs. ever	1.40	0.29–6.67	0.67	1.62	0.23–11.6	0.63
PS						
0–1 vs. 2–4	1.01	0.32–3.61	0.91	0.77	0.2–2.92	0.70
Thoracic RT						
No vs. yes	0.78	0.24–2.61	0.69	1.26	0.33–4.82	0.73
Pre‐existing IP						
No vs. yes	12.6	2.46–61.8	**0.002**	14.7	2.16–99.6	**0.006**

IP, interstitial pneumonia; ORs, odds ratios; PS, performance status; RT, radiotherapy.

## Discussion

In this retrospective analysis, patients who developed ILD had a favorable ORR and PFS compared with patients without ILD. Moreover, the results of multivariate analysis revealed that development of ILD and good PS were independent predictors for a favorable PFS. Recent reports have suggested a correlation between the development of irAEs and the clinical efficacy of ICIs in various types of cancer.^10^, ^11^ In advanced NSCLC patients, two prospective studies revealed that irAEs patients had a significantly higher ORR and longer PFS than non‐irAEs patients.^12^, ^13^ Immune‐related thyroid dysfunction was significantly correlated with a longer OS.^14^ Development of cutaneous irAEs correlated with an improvement in PFS in melanoma patients treated with ICIs.^11^ In NSCLC, Fujimoto *et al*. showed the ORRs were higher in patients with ILD than in those without (37% vs. 18%, respectively) and the PFS was significantly longer (5.8 vs. 2.1 months, respectively; *P* = 0.002).^15^ We also found a positive correlation between the development of ILD and clinical efficacy. Moreover, we report the details and risk factors of ILD. Recently, Nishino *et al*. reported two cases of nivolumab‐induced ILD in advanced NSCLC patients, demonstrating a radiographic pattern of cryptogenic organizing pneumonia (COP).^16^ Kato *et al*. reported seven of eight patients had a pattern of OP or nonspecific interstitial pneumonia.^17^ Naidoo *et al*. also showed an ILD pattern for COP, with ground‐glass opacities (GGO), interstitial involvement, and hypersensitivity. The histopathological findings were reported as cellular interstitial pneumonitis and OP.^18^ In this study, the radiologic pattern of ILD was OP (56%), DAD (13%), HP (6%) and NOS (25%). The DAD pattern was associated with a poor prognosis and high mortality in various cases.^19^ In our research, two of three patients who developed grade 5 ILD showed the DAD pattern. Furthermore, the OP pattern had a favorable prognosis compared with the non‐OP pattern. Therefore, these findings might help predict the prognosis of ILD.

Treatment strategies for immunotherapy‐induced ILD have been reported.^20^ Drug withdrawal is required for most patients with all grades of pneumonitis, and patients with grade 2 or higher are considered for treatment with oral/intravenous corticosteroids. In our study, 12 patients received steroid therapy and four patients were observed without any medication. Although steroid pulse and cyclophosphamide administration were provided, three of 16 patients died. The median time to the development of ILD was 28 days. Myriam *et al*. reported that the median time to the onset of development of ILD was 2.3 months.^21^ However, Suresh *et al*. reported that cases of grade ≥3 ILD occurred earlier than lower‐grade ILD.^22^ Therefore, careful monitoring of any respiratory symptom is important during ICI treatment, especially within 1–2 months.

Identifying risk factors for ILD is also important. In an interim analysis based on a data set of 1005 Japanese patients, reported risk factors for the occurrence of ILD were: age (≥75 years), abnormal chest CT findings other than lung cancer, and treatment line (previously treated). We identified pre‐existing IP as an independent risk factor for ICI‐related ILD. In our study, seven patients with pre‐existing IP were treated with ICI, and four patients developed ILD. Two of the four patients died because of ILD. Kanai *et al*. also showed that the incidence and severity of ILD were significantly higher in those with pre‐existing IP.^23^ Therefore, further careful monitoring for ILD is needed during ICI treatment in patients with pre‐existing IP.Recently, Tanaka *et al*. examined the characteristics of CD8^+^ T cells in peritumoral pleural effusion and BALF from nivolumab‐induced ILD in metastatic kidney cancer patients. The expression pattern of CD8^+^ T cells overlapped and they concluded that ILD was induced by nivolumab‐activated peritumoral CD8^+^ T cells.^24^ Laubli *et al*. also revealed a similarity between T cells in irAEs lesions and tumor infiltrating lymphocytes (TILs).^25^ These findings suggest that ILD reflects the infiltration of T cells with a specificity similar to tumor‐infiltrating T cells. In our study, lymphocytes were predominant and CD8 T cells were significantly elevated in the BALF in three of four patients. Although these immune cells might directly attack primary lung tumors, they also damage normal lung tissues, which might aid the development of ILD. This theory might account for the strong correlation between ICI‐related ILD and efficacy.

This analysis had several limitations. First, this was a single institution and retrospective study. Second, the PD‐L1 status of tumors assessed by immunostaining was not analyzed in all patients because a PD‐L1 immunostaining diagnostic kit was not approved in Japan until February 2017. Therefore, we could not evaluate PD‐L1 status as a risk factor for ILD. Indeed, in 16 patients with ILD, seven (44%) patient samples showed strong PD‐L1 expression. Therefore, we could not completely exclude that strong PD‐L1 expression affected good prognosis and occurrence of ILD. However, in Fig S2a,b patients with ILD showed almost the same prognosis (median PFS 15.9 months). Therefore, patients with ILD might have a good prognosis regardless of PD‐L1 expression. Third, to avoid guarantee‐time bias due to a higher chance of ILD patients with increasing treatment with ICIs, landmark analysis was conducted. However, the sample size was small, and only 16 patients demonstrated ILD; therefore, landmark analysis was not conducted.

In summary, we found that ICI‐induced ILD was associated with efficacy in NSCLC and pre‐existing ILD was a risk factor for ILD. Further studies are needed to clarify the mechanism between ILD and clinical response.

## Disclosure

Masahiro Seike, Kaoru Kubota, and Akihiko Gemma receive an honorarium from Merck Sharp & Dohme and Bristol‐Myers Squibb. The authors report no other conflicts of interest in this work.

## Supporting information


**Table S1** Objective response rates to immune checkpoint inhibitors.
**Table S2** Types of immune related adverse events.

**Figure S1** Rate of progression‐free survival (PFS) in the study population. Kaplan‐Meier curves are shown for progression‐free survival. (**a**) Median PFS for untreated patients. (**b**) Median PFS for previously treated patients; red line.
**Figure S2** Rate of progression‐free survival (PFS) in the study population. Kaplan‐Meier curves are shown for progression‐free survival. (**a**) Median PFS for untreated patients. (**b**) median PFS for previously treated patients; red line; ILD, green line; irAEs‐non‐ILD, black line; non‐irAEs. Abbreviations: irAEs, immune‐related adverse events; ILD, interstitial lung disease; NR, not reached; NA, not available.
**Figure S3** Computed tomography of the chest shows organizing pneumonia (OP) pattern (**a**) and diffuse alveolar damage (DAD) pattern (**b**).
**Figure S4** Rate of progression‐free survival in the study population. Kaplan‐Meier curves are shown for progression‐free survival, red line, OP pattern; black line, non‐OP pattern. Abbreviations: OP, organizing pneumonia; NA, not available.
