# Supplementary material for: Immune checkpoint inhibitor‐associated interstitial lung diseases correlate with better prognosis in patients with advanced non‐small‐cell lung cancer
Source: Thorac Cancer. 2020 Feb 25;11(4):1052–60. doi: 10.1111/1759-7714.13364 (PMC7113045; doi:10.1111/1759-7714.13364)
Supplement: Supplementary file 1 — Table S1 Objective response rates to immune checkpoint inhibitors. Table S2 Types of immune related adverse events. [file TCA-11-1052-s001.docx]

**Supplementary Table 1**

Objective response rates to immune checkpoint inhibitors

| **Response,** | **Overall**  **(*n* = 130)** | **ILD group**  **(*n* = 16)** | **irAEs-non-ILD group (*n* = 23)** | **Non-irAEs**  **group (*n* = 91)** | ***P-*value** |
| --- | --- | --- | --- | --- | --- |
| CR, *n*, (%) | 0 (0) | 0 (0) | 0 (0) | 0 (0) |  |
| PR, *n*, (*%*) | 39 (30) | 10 (63) | 10 (43) | 19 (21) | <0.0001 |
| SD, *n*, (%) | 36 (27) | 2 (12) | 6 (26) | 28 (30) |  |
| PD, *n*, (%) | 40 (31) | 2 (12) | 4 (17) | 34 (37) |  |
| NE, *n*, (%) | 15 (11) | 2 (12) | 3 (13) | 10 (11) |  |

Abbreviations: irAEs, immune related adverse events; ILD, interstitial lung disease; CR, complete response; PR, partial response; SD, stable disease; PD, progressive disease; NE, not evaluable

**Supplementary Table 2**

Types of immune related adverse events

| **Immune related**  **adverse event** | **Any grade**  ***n* (%)** | **≥ Grade 3**  ***n* (%)** |
| --- | --- | --- |
| All irAEs | 39 (30) | 15 (11) |
| ILD | 16 (12) | 11 (8.3) |
| Hypothyroidism | 9 (6.8) | 0 (0) |
| Skin toxicity | 5 (3.8) | 0 (0) |
| Nephrotoxicity | 3 (2.2) | 1 (0.8) |
| Encephalitis | 3 (2.2) | 3 (2.2) |
| Hepatotoxicity | 2 (1.5) | 1 (0.8) |
| Adrenal insufficiency | 2 (1.5) | 0 (0) |
| Cardiotoxicity | 1 (0.8) | 1 (0.8) |
| Type I diabetes mellitus | 1 (0.8) | 1 (0.8) |
| Myasthenia Gravis | 1 (0.8) | 0 (0) |

Abbreviations: irAEs, immune related adverse events; ILD, interstitial lung disease
