# Supplementary material for: Immune checkpoint inhibitor‐associated interstitial lung diseases correlate with better prognosis in patients with advanced non‐small‐cell lung cancer
Source: Thorac Cancer. 2020 Feb 25;11(4):1052–60. doi: 10.1111/1759-7714.13364 (PMC7113045; doi:10.1111/1759-7714.13364)
Supplement: Supplementary file 2 — Figure S1 Rate of progression‐free survival (PFS) in the study population. Kaplan‐Meier curves are shown for progression‐free survival. (a) Median PFS for untreated patients. (b) Median PFS for previously treated patients; red line. Figure S2 Rate of progression‐free survival (PFS) in the study population. Kaplan‐Meier curves are shown for progression‐free survival. (a) Median PFS for untreated patients. (b) median PFS for previously treated patients; red line; ILD, green line; irAEs‐non‐ILD, black line; non‐irAEs. Abbreviations: irAEs, immune‐related adverse events; ILD, interstitial lung disease; NR, not reached; NA, not available. Figure S3 Computed tomography of the chest shows organizing pneumonia (OP) pattern (a) and diffuse alveolar damage (DAD) pattern (b). Figure S4 Rate of progression‐free survival in the study population. Kaplan‐Meier curves are shown for progression‐free survival, red line, OP pattern; black line, non‐OP pattern. Abbreviations: OP, organizing pneumonia; NA, not available. [file TCA-11-1052-s002.pptx]

## Slide 1
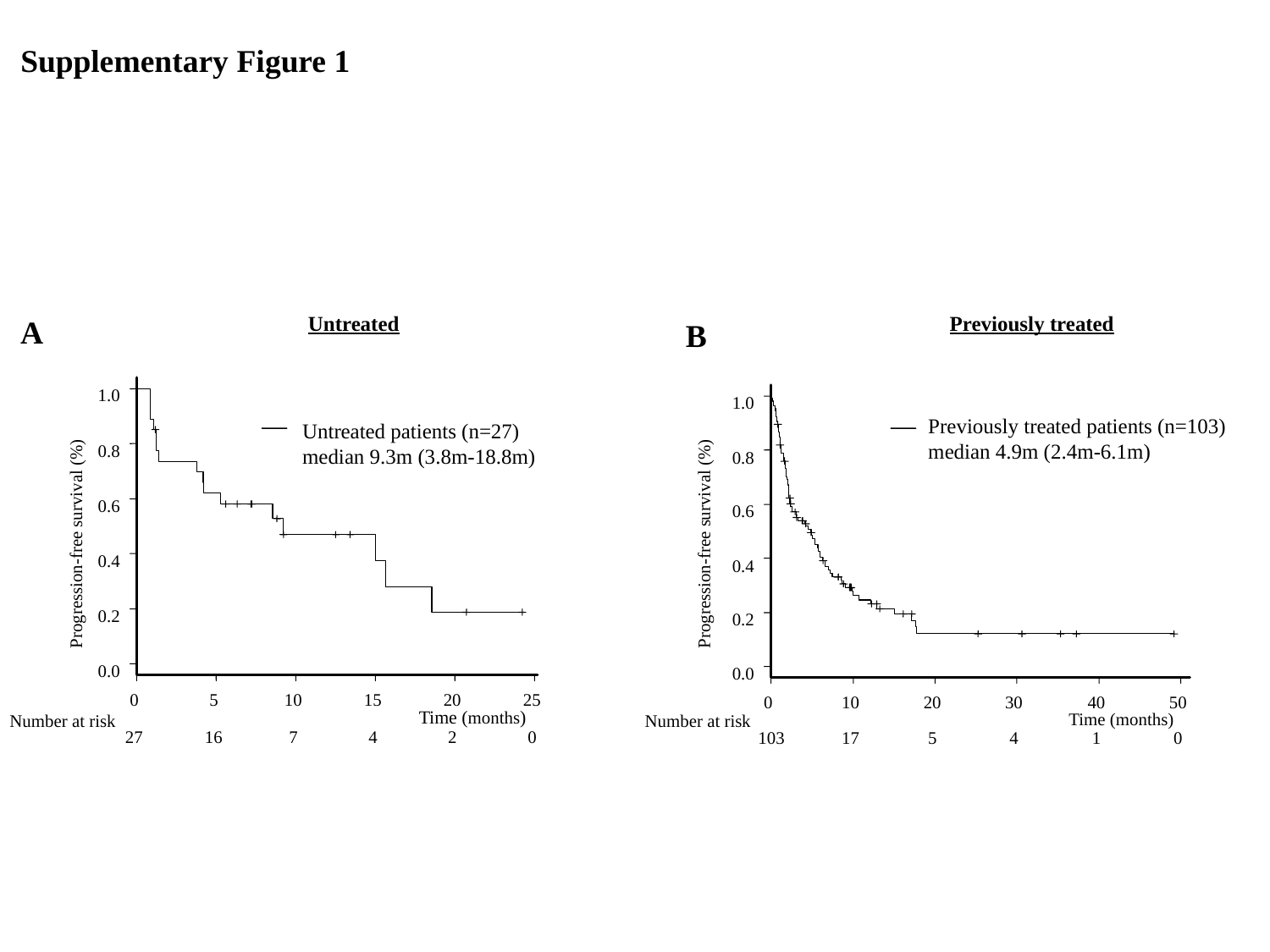

Supplementary Figure 1
Untreated
Previously treated
A
B
1.0
0.8
0.6
0.4
0.2
0.0
0
5
10
15
20
25
Number at risk
27
16
7
4
2
0
1.0
0.8
0.6
0.4
0.2
0.0
0
10
20
30
40
50
Number at risk
17
5
4
1
0
103
Previously treated patients (n=103)
median 4.9m (2.4m-6.1m)
Untreated patients (n=27)
median 9.3m (3.8m-18.8m)
Progression-free survival (%)
Progression-free survival (%)
Time (months)
Time (months)

## Slide 2
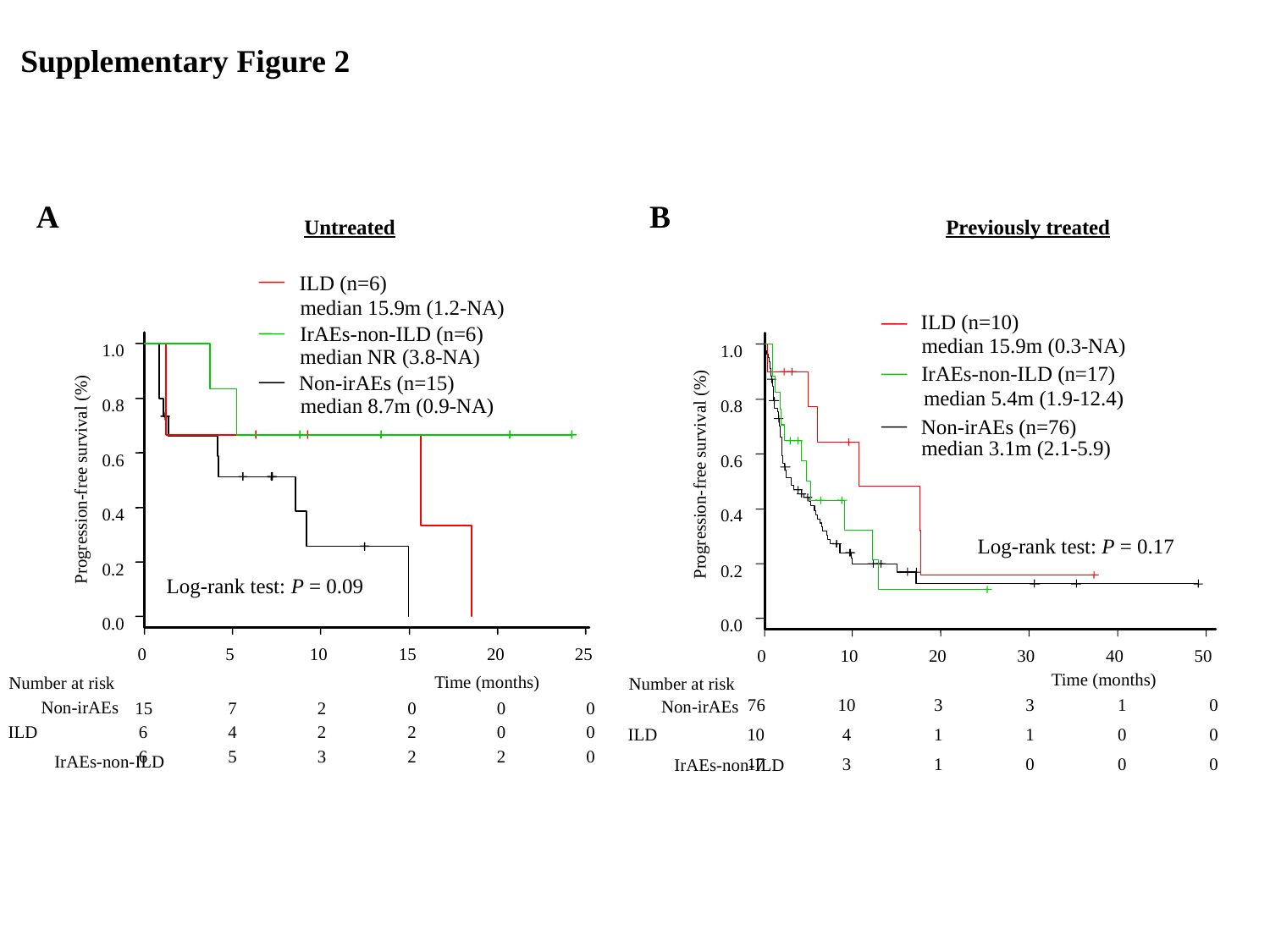

Supplementary Figure 2
A
B
Untreated
Previously treated
ILD (n=6)
median 15.9m (1.2-NA)
ILD (n=10)
IrAEs-non-ILD (n=6)
median 15.9m (0.3-NA)
1.0
0.8
0.6
0.4
0.2
0.0
0
5
10
15
20
25
1.0
0.8
0.6
0.4
0.2
0.0
0
10
20
30
40
50
median NR (3.8-NA)
IrAEs-non-ILD (n=17)
Non-irAEs (n=15)
median 5.4m (1.9-12.4)
median 8.7m (0.9-NA)
Non-irAEs (n=76)
median 3.1m (2.1-5.9)
Progression-free survival (%)
Progression-free survival (%)
Log-rank test: P = 0.17
Log-rank test: P = 0.09
Time (months)
Time (months)
Number at risk
Number at risk
76
10
3
3
1
0
10
4
1
1
0
0
17
3
1
0
0
0
Non-irAEs
ILD
IrAEs-non-ILD
Non-irAEs
15
7
2
0
0
0
6
4
2
2
0
0
6
5
3
2
2
0
ILD
IrAEs-non-ILD

## Slide 3
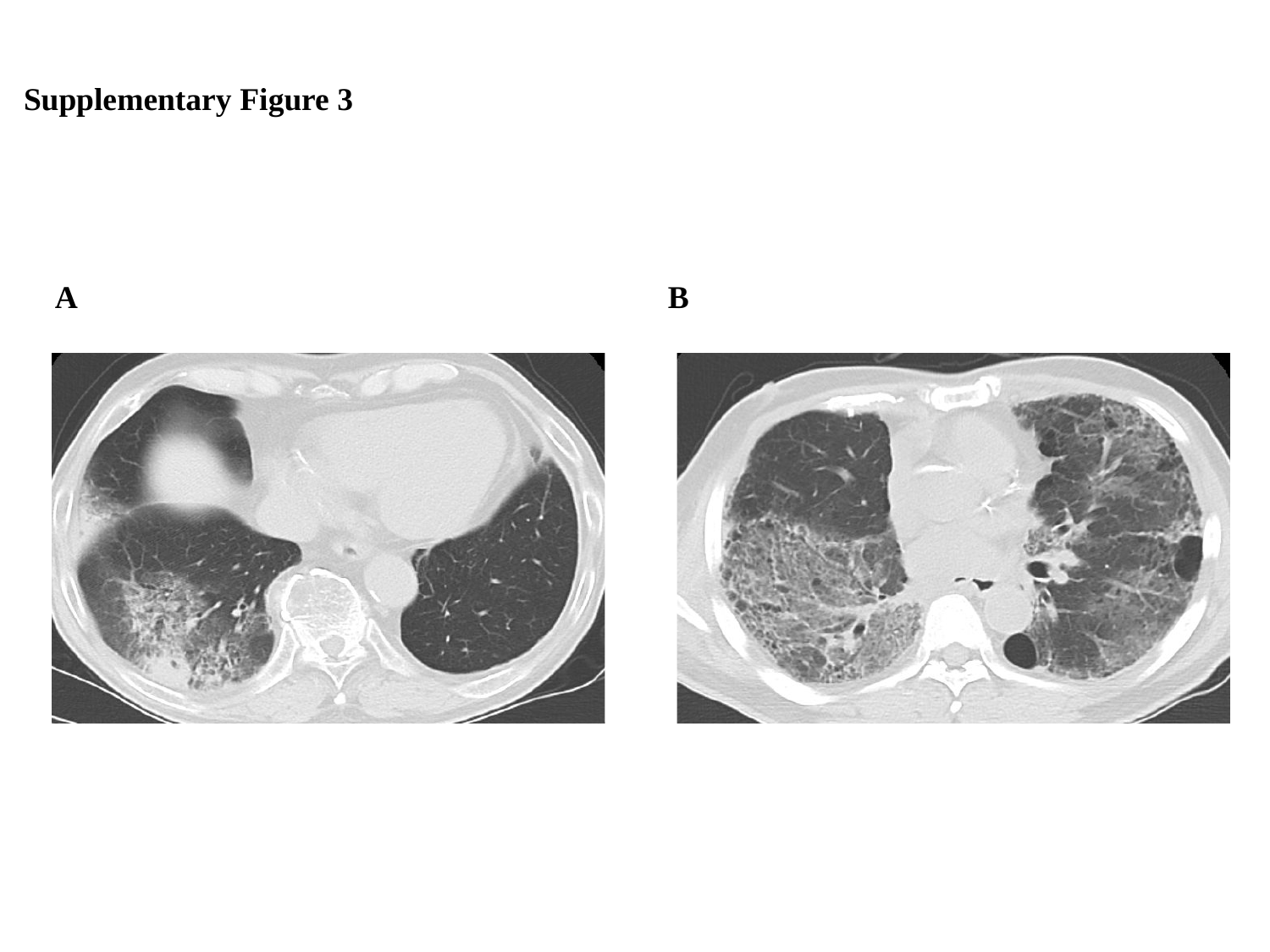

Supplementary Figure 3
A
B

## Slide 4
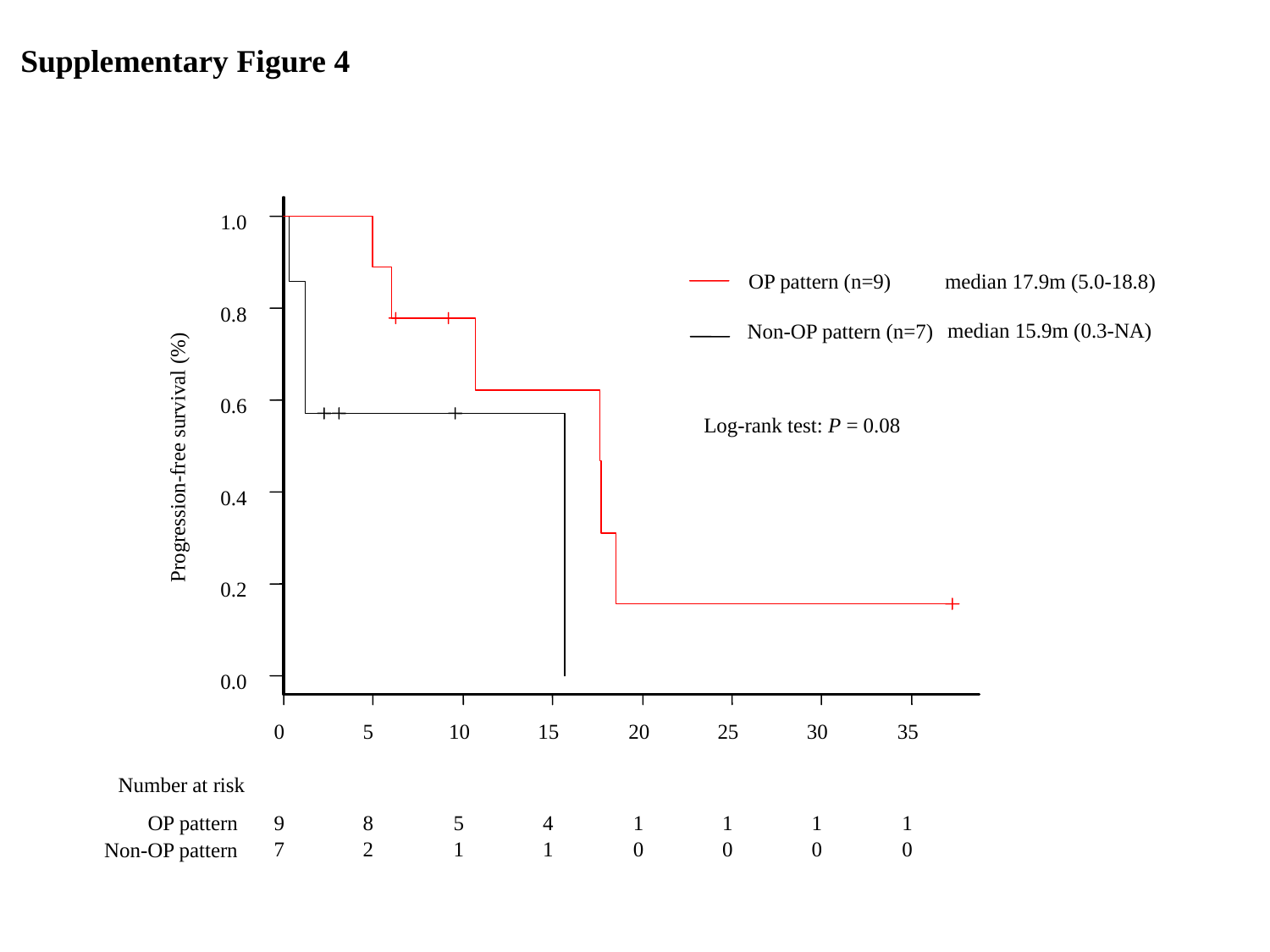

Supplementary Figure 4
1.0
OP pattern (n=9)
median 17.9m (5.0-18.8)
0.8
median 15.9m (0.3-NA)
Non-OP pattern (n=7)
0.6
Log-rank test: P = 0.08
Progression-free survival (%)
0.4
0.2
0.0
0
5
10
15
20
25
30
35
Number at risk
9
8
5
4
1
1
1
1
OP pattern
7
2
1
1
0
0
0
0
Non-OP pattern
